# Late-Induced Autoimmune Disorders Post-COVID-19 Vaccination/Infection: Case Report From Iran

**DOI:** 10.1155/carm/8815875

**Published:** 2025-08-28

**Authors:** Mona Sadat Larijani, Anahita Bavand, Fatemeh Ashrafian, Ladan Moradi, Amitis Ramezani

**Affiliations:** Clinical Research Department, Pasteur Institute of Iran, Tehran, Iran

**Keywords:** autoimmune diseases, COVID-19, systemic lupus erythematosus, thyroiditis, vaccines

## Abstract

COVID-19 pandemic led to a fast vaccine design due to the threat of rapid spreading worldwide. Safety profile of the approved vaccines has been achieved mostly through clinical trials. However, some unsolicited adverse events in a longer duration of time have been recorded in addition to the late disorders known as long-COVID, stemming from classical infection. Therefore, case studies and long-term follow-up are required to enrich the current data on SARS-COV-2 infection/vaccination. In this study, two cases of autoimmune diseases induced by COVID-19 and/or vaccination were followed in three years. The profile of each is presented, and the probable cause has been discussed. The laboratory findings approved systematic lupus erythematosus and Hashimoto's thyroiditis in the studied cases. The key finding of this study is that the importance of probable autoimmune diseases flares up in individuals with a history of autoimmunity in their families which could manifest as a long-COVID symptom or late vaccination side effect.

## 1. Introduction

Viral infection could possibly modify the induction of autoimmune disorders. It is suggested that viral-induced autoimmunity can be triggered through different mechanisms such as modified cell cycles of infected B-cells and molecular mimicry [1, 2]. In addition, autoimmunity linked to COVID-19 infection and/or vaccination has been reported in some studies [3–5]. COVID-19 vaccines have been administered widely to prevent the severe form of the disease. Booster doses have been then recommended due to the fact that the effectiveness of vaccines generally declines over time [6, 7]. There is a growing level of information regarding long-term adverse events including case reports with new-onset of a condition or relapsed autoimmune diseases following COVID-19 and its vaccines [8–10]. Late onset refers to the emergence of signs or symptoms of a medical condition occurring after a defined latency period postvaccination, typically beyond the expected window for common acute adverse events, which is generally considered to be more than 7–14 days after vaccination, depending on the vaccine and the condition. Late onset is usually considered for more than 21–42 days (3–6 weeks) after COVID-19 vaccination. In vaccine safety monitoring, “early onset” is normally defined within 0–7 or 0–21 days, and events beyond 21 or 42 days are considered late [11–13]. This definition has been added to the manuscript.

Nonetheless, the relationship between the progression of autoinflammatory diseases and classical infection or vaccination remains controversial. Hereby, we report two cases, a mother and daughter, with induced autoimmune diseases after COVID-19 and/or vaccination against it.

## 2. Case Presentation

**Figure 1:**
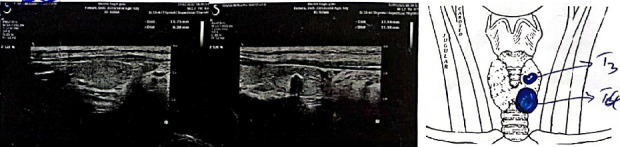
Color Doppler sonography of the thyroid gland and neck. The findings indicated that both thyroid lobes have small size and heterogeneously decreased parenchymal echo suggesting chronic lymphocytic thyroiditis. Two focal lesions at the left lobe were seen: N1: an 18 ∗ 13 ∗ 11.7-mm well-defined isoechoic solid nodule contains coarse and fine calcifies foci and border line elastoscan is seen at lower part (TRIDAS Iva, moderately suspicious). N2: an 11.7 ∗ 6.3 ∗ 7.8-mm well-defined isoechoic without calcification with peripheral vascularity and soft elasticity as elastoscan is seen at the junction of isthmus to the left lobe (TIRADS III, mildly suspicious).

A 64-year-old woman without any history of underlying diseases sought medical care due to the swollen and painful neck. Through the medical examination, she also complained about hair loss, constipation, and weight gain. After physical examination by the endocrinologist, laboratory tests (Table 1) and sonography, which indicated two nodules at the left lobe of the thyroid gland, were performed (Figure 1). She subsequently underwent the thyroid biopsy. No malignancy was detected in the biopsy, and the results indicated Hashimoto's thyroiditis. She started to take levothyroxine 50 mg daily.

During the follow-up, she felt morning stiffness in her metacarpophalangeal (MCP) and proximal interphalangeal (PIP) joints. This symptom started almost 10 months after Hashimoto's thyroiditis diagnosis. Therefore, laboratory tests for autoimmune disorders were performed (Table 2). According to the results and based on the case description, considering a positive familial history (the patient's brother suffers from lupus nephritis), systemic lupus erythematosus (SLE) diagnosis was considered in this case. Eventually, she was recommended to take hydroxychloroquine 250 mg and prednisolone 5 mg and coenzyme Q10, every day. During the follow-up, her symptoms have significantly improved, and accordingly hydroxychloroquine 250 mg and prednisolone 5 mg were reduced to be taken twice a week.

During the COVID-19 vaccination program, she got the first dose of Sinopharm vaccine on July 5th, 2021, followed by the second dose 28 days later. Furthermore, she received a booster dose of PastoCovac Plus on January 9th, 2022. Her medical history showed a history of COVID-19 after vaccination in February, 2022, with mild symptoms including cough, body pain, and headache which were resolved after seeking supportive medical care.

**Figure 2:**
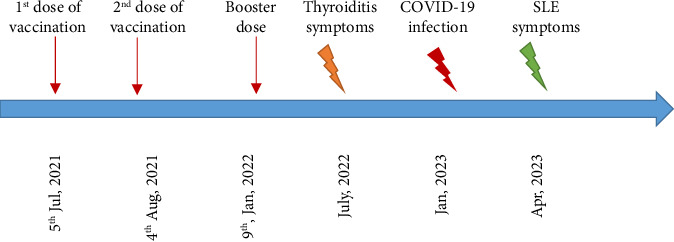
The schematic view of the COVID-19 incidence and its vaccination along with the late disorders in Case 1.

According to the investigated medical history summarized in Figure 2, Hashimoto's thyroiditis initiated almost 5 months after the booster dose, whereas SLE symptoms were experienced about 4 months after COVID-19 infection. Therefore, it seems that COVID-19 vaccine could be a coeffector to the thyroid disorder as a late new onset, while SLE seems to be triggered by classical infection in the form of long-COVID.

Neck survey showed no pathologic lymph node at all neckzones and retrosternal area all visible lymph nodes show normal hilum and color Doppler appearance. Both thyroid lobess howed small size and heterogeneously decreased echo suggesting chronic lymphocytic thyroiditis (Hashimoto + left T3 and T4 nodules).

The second case, the first patient's daughter, a 36-year-old woman without any underlying diseases, referred to the medical center with a pain in the neck and swollen thyroid gland. She also complained of hair loss. According to her familial history and symptoms, she underwent laboratory tests and sonography in which the results were in favor of Hashimoto's thyroiditis (Table 3). She was recommended to take levothyroxine 50 mg daily. Her symptoms have significantly improved after 6 months.

She received her first dose of COVID-19 vaccine, Covaxin, on May 27th, 2021, followed by the second dose 28 days later. She also got a booster of PastoCovac Plus on October 3rd, 2021. Her medical history also showed a positive COVID-19 history a year after vaccination with mild symptoms of fever and fatigue. She did not get any medical care for COVID-19.

**Figure 3:**
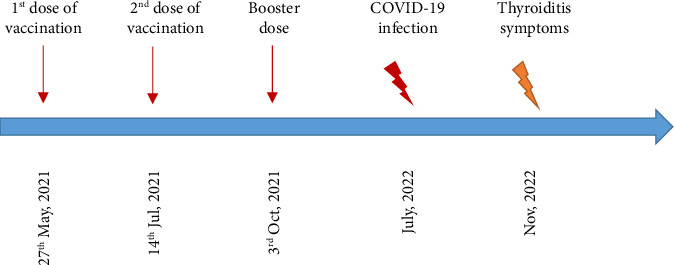
The schematic view of the COVID-19 incidence and its vaccination along with the late thyroiditis symptoms in Case 2.

Based on her medical profile illustrated in Figure 3, Hashimoto's thyroiditis symptoms started about four months post-COVID-19 infection and seem to be triggered by SARS-CoV-2 rather than vaccination. She was recommended to take levothyroxine 50 μg per day which led to releasing the pain and swelling 1 week after consumption.

## 3. Discussion

Viral infections have been considered as crucial environmental inducers of autoimmune disease. This issue is more relevant regarding COVID-19 pandemic, as millions of individuals have been infected with SARS-CoV-2 and also got vaccinated against it. Although there have been several case reports of autoimmune diseases new onsets so far, there is still the lack of cumulative data in the context overall risk of autoimmune disease after infection [8, 14]. From another point of view, vaccination against COVID-19 has been undoubtedly proven to be effective in prevention from severe infection and the rate of mortality. However, there are emerging reports and case studies which propose that these vaccines might induce autoimmune diseases, including autoimmune hepatitis and thyroiditis [15, 16]. Although there are concerns over unsolicited vaccine side effects with the potency of public hesitancy about vaccination, the causal relationship between the applied vaccines and the phenomena of autoimmune disorders requires more investigation. It has been shown that vaccines could trigger autoimmunity through different mechanisms including anti-idiotypic network, epitope spreading, and molecular mimicry [17]. Moreover, COVID-19 infection itself has been shown to induce autoimmunity in forms of Guillain–Barré syndrome (GBS), rheumatoid arthritis, hemolytic anemia (AIHA), and thrombocytopenic purpura [18]. The mechanism through which an autoimmune disease develops after COVID-19 infection is still unclear, though it has been proposed that cases with a history of severe form of COVID-19 would have increased B-cell activation which leads to enhanced antibody secreting cell lines and therefore increased immune responses [19]. Several thyroid disorders have been detected following COVID-19 infection or immunization [20, 21]. Hashimoto's thyroiditis is an autoimmune disorder in which immune-system activity leads to a decline in hormone production [22]. According to the literature review, all COVID-19 vaccines have the potency to induce a kind of thyroid disorder ranging from 1 to almost 90 days postimmunization, though the investigation on mRNA vaccines have been dominant [23]. In the present study, both the cases have been immunized through a heterologous vaccine regimen including inactivated and protein vaccines. Case No. 1 had an episode of COVID-19 infection 1 year after the booster dose, and the thyroiditis symptoms started 6 months after booster dose and before the COVID infection. Nevertheless, Case 2 presented the symptoms of thyroid abnormality nearly 4 months after the COVID infection. Therefore, it seems that both infection and vaccination could induce to late onset of Hashimoto's thyroiditis which could be considered as long-COVID or long-vaccine manifestations.

A cohort study performed in 2021 by 122 patients was carried out to monitor the thyroid autoimmunity post-COVID-19. Similar to our findings, anti-TPO increased 3 months after acute phase of infection [24]. A case study also presented a woman with fatigue and severe hair loss 20 days post-COVID infection. The sonographic examination showed the heterogeneous nodule in the thyroid gland [25]. Although the symptoms such as fatigue and hair loss might be similar to long-COVID-19 disorder, it is crucial for clinicians to consider new onset of thyroid dysfunction and follow the cases through related diagnostic tests. In a study by Zhong et al., five cases of thyroiditis were reported after COVID vaccination, in which patients experienced symptoms, including neck pain, initiated 2–44 days after receiving inactivated COVID vaccines. Nevertheless, they did not have any history of COVID-19 before the vaccination or at the time of admission [26].

In a review on 98 subjects with COVID-19 vaccine-induced thyroiditis it has been declared that although there are similarities between SARS-CoV-2 induced thyroiditis and the vaccine-induced ones, this autoimmune disorder is more common in young and middle-aged cases and also is dominant in women rather than men, which is in line with our presented cases. Furthermore, in agreement with our report, the vast majority of the cases did not have any pre-existing thyroid disorder and nearly all the cases suffered from local neck pain and swelling [21].

SLE is a chronic autoimmune disease which could affect a wide range of organs. Lupus occurs through autoimmune activity though its precise mechanism remains unclear [27, 28]. New onset of SLE triggered by COVID-19 vaccination have been detected in the literature, mostly detected in a short-time period after vaccination ranging from one day to six weeks [29, 30]. SLE was reported following different types of COVID-19 vaccines including mRNA, inactivated, and viral vector ones [31]. In this study, we report a case of SLE whose symptoms initiated almost 2 years postvaccination of a heterologous regimen including inactivated and protein vaccine platform. Considering the duration between vaccination and SLE symptoms, it is less probable to be induced by the applied vaccines. However, she had an episode of COVID-19 infection four months before the symptoms initiation. According to the familial history of SLE, COVID-19 infection could act as a trigger to the autoimmune reaction.

In a case-report study by Ramachandran et al., a patient complained about bilateral lower extremity swelling and shortness of breath in addition to fatigue and malaise 1 month post-COVID infection in which the laboratory and physical evaluation indicated him as a case of acute renal failure and the kidney biopsy subsequently confirmed SLE [32]. Another study presented a patient who experienced scaling on the palms and soles, ankle swelling, and lower extremity edema 2 months after SARS-CoV-2 infection in which the laboratory tests and physical examination indicated a positive case of SLE [33].

The reported SLE cases post-COVID-19 have been captured mostly within two months postrecovery. The reported case in this study, presented a late onset of SLE almost four months after COVID-19 infection. It has been shown that antinuclear autoantibodies (ANAs) could be detected up to 12 months postacute COVID infection. These antibodies are able to target cell nuclei-promoting inflammation components and subsequently damage organ systems [34]. Therefore, the durability of the related antibodies might be a trigger to autoimmunity in late onset of disorders.

Although the current evidence is still limited regarding the differences in clinical characteristics between COVID-19 infection and SARS-CoV-2 vaccine-induced autoimmune diseases, a direct comparison study has shown that the symptoms and duration of treatment regarding thyroid disorders were more limited in vaccine-induced cases than the classical infection-induced ones [21]. There could be an association between the autoimmune incidences and COVID-19 infection or vaccination against it; however, the discussed disorders might only stem from host genetics factor, age, familial history, and lifestyle. In other words, the casual relationship between the late onset of autoimmune disease is a challenge which needs more investigation and long-term follow-up studies. Therefore, the associated studies must be conducted to record new cases, and this evidence should be subsequently categorized in a set of cumulative data to achieve satisfying results. Nevertheless this study is limited to reporting Hashimoto's thyroiditis as an autoimmune disease which is more prevalent and documented after viral illnesses such as COVID-19. Although no previous condition was recorded in the cases' medical profiles and only a familial history of SLE was documented, it seems that COVID-19 vaccination could trigger autoimmunity as well as the infection itself to flare up the late symptoms. However, considering a causal relationship between the late symptoms and vaccination and/or infection is not fully possible based on the current knowledge and published data. This report accompanied by other case series could provide lines of evidence for longitude studies and follow-up schedule on larger populations to conclude a more precise association.

What makes this report remarkable is the potential act of the virus and/or its vaccination in triggering autoimmune diseases of the population with a positive familial background as a late manifestation. The cumulative data would significantly contribute to the present knowledge over the role of recent pandemic and also probable future pandemic in the context of pathogens and their immunization as possible causes of autoimmunity. Moreover, it seems rational to investigate autoimmune diseases flare up among families with a history of autoimmunity to estimate the probability of COVID-19 infection/vaccination in association with this issue, not only as an immediate side effect but also as a possible late unsolicited adverse event.

## Figures and Tables

**Table 1:** Thyroid function tests of Case 1.

Test	Result	Unit	Normal range
Antithyroid peroxidase (anti-TPO)	> 1000	IU/mL	Up to 40
T4	109.07	nmol/L	58.1–140.6
T3 up	0.94	TBI	0.8–1.3
FT1	0.9	μg/dL	4.8–12.7
T3	1.6	nmol/L	0.92–2.79
TSH	1.93	μIU/mL	0.55–4.78

**Table 2:** SLE associated laboratory findings of Case 1.

Test	22 May, 2023	Unit	Normal range	19 July, 2023	Normal range	Unit	21 September, 2023	Normal range	Unit
CRP	0.4	mg/L	Up to 6.0	0.4	Up to 6.0	mg/L	0.41	Up to 6.0	mg/L

RF	8.90	IU/mL	Up to 20	—	Up to 6.0	IU/mL	—	Up to 6.0	IU/mL

ANA	1.54	Index	Negative < 1.0	—	Negative < 1.0	Index	—	Negative < 1.0	Index
			Positive:≥ 1.0		Positive:≥ 1.0			Positive:≥ 1.0	

Anti-dsDNA	287.0	IU/mL	Negative: < 30.0	42.5	Negative: < 30.0	IU/mL	—	Negative: < 30.0	IU/mL
			Borderline: 30–35		Borderline: 30–35			Borderline: 30–35	
			Positive: > 35.0		Positive: > 35.0			Positive: > 35.0	

Anti-DsDNA IgG	—	IU/mL	Negative: < 100	42.5	Negative: < 100	IU/mL	32.2	Negative: < 100	IU/mL
			Positive:≥ 100		Positive:≥ 100			Positive:≥ 100	

Anti-CCP	—	U/mL	Negative: < 30	5.5	Negative: < 30	U/mL	—	Negative: < 30	U/mL
			Positive:≥ 30		Positive:≥ 30			Positive:≥ 30	

Anti-SS-A (anti-RO)	—	U/mL	Negative: < 12	15	Negative: < 12	U/mL	—	Negative: < 12	U/mL
			Borderline: 12–18		Borderline: 12–18			Borderline: 12–18	
			Positive: > 18		Positive: > 18			Positive: > 18	

Abbreviations: ANA = antinuclear antibodies, Anti-CCP = anticyclic citrullinated peptide antibodies, CRP = C-reactive protein, ds-DNA = double-stranded DNA, FTI = thyroxin index, RF = rheumatoid factor, and T3 Up = T3 uptake.

**Table 3:** Laboratory results of Case 2.

Test	Antithyroid peroxidase (anti-TPO)	Normal range	Unit
Antithyroid peroxidase (anti-TPO)	302.2	Upto 35	IU/mL
T3	1.47	1.3–3.1	nmol/mL
T4	8.42	5.1–14.1	μg/dL
TSH	2.45	0.27–4.2	mIU/L

## Data Availability

All the data are included in the manuscript.

## Supporting Information

CARE checklist of information is presented in the supporting file.
