# Supplementary material for: Late-Induced Autoimmune Disorders Post-COVID-19 Vaccination/Infection: Case Report From Iran
Source: Case Rep Med. 2025 Aug 28;2025:8815875. doi: 10.1155/carm/8815875 (PMC12411051; doi:10.1155/carm/8815875)
Supplement: Supporting Information — Additional supporting information can be found online in the Supporting Information section. [file 8815875.f1.docx]

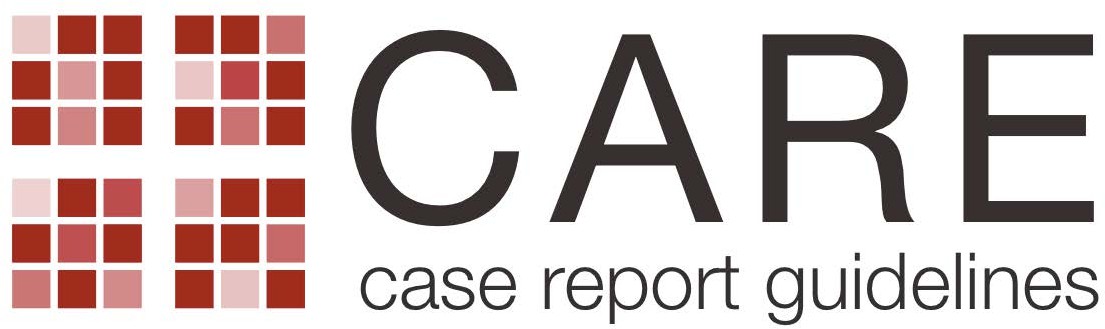
CARE Checklist of information to include when writing a case report
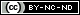


**Topic Item Checklist item description Reported on Line**

**Title 1** The diagnosis or intervention of primary focus followed by the words “case report” . . . . . . . . . . . . . . . . . . 2

**Key Words 2** 2 to 5 key words that identify diagnoses or interventions in this case report, including "case report" 30

**Abstract**

**(no references)**

**3a** Introduction: What is unique about this case and what does it add to the scientific literature? 19-22

**3b** Main symptoms and/or important clinical findings . . . . . . . . . . . . . . . . . . . . . . . . . . . . . . . . . . . . . . . . . . . . . . . . . . . 23-25

3c The main diagnoses, therapeutic interventions, and outcomes 25-27

3d Conclusion—What is the main “take-away” lesson(s) from this case? 28-30

Introduction 4 One or two paragraphs summarizing why this case is unique (may include references) 36-48

Patient Information 5a De-identified patient specific information 50

5b Primary concerns and symptoms of the patient 51-52

5c Medical, family, and psycho-social history including relevant genetic information 88-94,119-122

5d Relevant past interventions with outcomes 62-65-114-116

Clinical Findings

Timeline

Diagnostic Assessment

Therapeutic Intervention

Follow-up and Outcomes

1. Describe significant physical examination (PE) and important clinical findings 110-122
2. Historical and current information from this episode of care organized as a timeline Yes ✓

8a Diagnostic testing (such as PE, laboratory testing, imaging, surveys). 62-65-114-116

8b Diagnostic challenges (such as access to testing, financial, or cultural) 62-65-114-1168c Diagnosis (including other diagnoses considered) 60-62,112-114

8d Prognosis (such as staging in oncology) where applicable not applicable

**9a** Types of therapeutic intervention (such as pharmacologic, surgical, preventive, self-care) . . . . . . . . . . . . . . . . . 62-65-114-116

**9b** Administration of therapeutic intervention (such as dosage, strength, duration) 62-65-114-116

**9c** Changes in therapeutic intervention (with rationale) not applicable

**10a** Clinician and patient-assessed outcomes (if available) 94-99

**10b** Important follow-up diagnostic and other test results 71-75

**10c** Intervention adherence and tolerability (How was this assessed?) 62-65

**10d** Adverse and unanticipated events not applicable

**Discussion 11a** A scientific discussion of the strengths AND limitations associated with this case report 128-202

**11b** Discussion of the relevant medical literature **with references** 128-202

**11c** The scientific rationale for any conclusions (including assessment of possible causes) 203-209

**11d** The primary “take-away” lessons of this case report (without references) in a one paragraph conclusion 210-217

**Patient Perspective 12** The patient should share their perspective in one to two paragraphs on the treatment(s) they received . . . . 210-217

**Informed Consent 13** Did the patient give informed consent? Please provide if requested . . . . . . . . . . . . . . . . . . . . . . . . . . . . . . . . . . . . . …..**Yes** ✓ **No
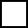
**
